# Supplementary material for: Agrivoltaics: Modeling the relative importance of longwave radiation from solar panels
Source: PLoS One. 2022 Oct 28;17(10):e0273119. doi: 10.1371/journal.pone.0273119 (PMC9616218; doi:10.1371/journal.pone.0273119)
Supplement: S1 File — (PDF) [file pone.0273119.s001.pdf]

In [1]:

```
%%time
import numpy as np
import math
import matplotlib.pyplot as plt

h0 = 1.1 # m
D = 6.2    # m
L = 3.56  # m
theta = 18*np.pi/180
sigma = 5.67*10**(-8) # W/m2-K4 ; Stephan Boltzmann constant
dl = 0.01 # m
T_panel = 300

# Make a function that takes x' as an input and returns q_shell
# EFFECT OF SINGLE PANEL ELEMENT

x_prime = np.linspace(0.1, L*np.cos(theta), 100)
xlim1 = h0 / np.tan(theta)
xlim2max = 212
#X = np.arange(-xlim1-xlim2max,xlim2max,0.2)
X = np.linspace(-xlim1-xlim2max,xlim2max,4000)

# x' will be a single value, and flux (q_shell) will be an array with a flux value at each x
def RadFlux(x_prime, h0, theta, D, T_panel, X):
    h = h0 + x_prime*np.tan(theta)

    xlim2 = -h * (x_prime - D)/(h-h0) + x_prime

    flux = dl*sigma* T_panel**4 / (math.pi*np.sqrt(h**2 + (X-x_prime)**2))

    # Set all fluxes to zero that are beyond xlim2
    for i in range(0,len(X)):
        if (X[i] - x_prime) > xlim2:
            flux[i] = 0

        if X[i] < -xlim1:
            if h > h0+0.1*np.tan(theta): # if the element is higher than the lowest element,
                flux[i] = 0              #   set all flux values beyond xlim1 to zero
    return flux, xlim2, X
```

```
CPU times: user 196 ms, sys: 78.7 ms, total: 275 ms
Wall time: 606 ms
```

In [2]:

```
%%time
# EFFECT OF ONE FULL PANEL
# Use RadFlux() to loop through all x_prime values, sum up all fluxes at each ground position x
# The resultant will be an array of fluxes, where each element is the TOTAL flux at a given x

def Flux_panel(h0, theta, D, T_panel, L, X):

    x_prime = np.linspace(0.1, L*np.cos(theta), int(L/dl))
    panelFluxes = np.zeros(len(X))

    for i in range(0,len(x_prime)):
        fluxes, xlim2, X = RadFlux(x_prime[i], h0, theta, D, T_panel, X)
        # ^Compute fluxes along ground from a given element at x'
        #plt.plot(X, fluxes)

        for n in range(len(fluxes)):
            panelFluxes[n] += fluxes[n]
            # Add the flux at each ground position to the total flux at that position
    return panelFluxes, X
```

```
CPU times: user 2 µs, sys: 0 ns, total: 2 µs
Wall time: 3.1 µs
```

In [3]:

```
%%time
# EFFECT OF ENTIRE SOLAR ARRAY
# Does Flux_panel() need to be called for each panel, if each panel has a unique temp,
# or can we generalize across all panels?

def ArrayFlux(h0, theta, D, T_panel, L, numpanels, X):

    PanelFluxes, X = Flux_panel(h0, theta, D, T_panel, L, X)
    delta_S = X[1]-X[0]

    S = np.arange(X[0],D*numpanels+X[-1],delta_S)  # Create giant array that includes all ground
points across
                                                    #  entire array, starting at leftmost xlim1
    ArrayFluxes = S*0  # empty mega-array to store final flux values
    n = D/delta_S # number of elements in S corresponding to one panel

    for i in range(0,numpanels):

        idx1 = int(i*n)                   # first index = ith panel * num ground elements per pane
l
        idx2 = int(i*n+len(PanelFluxes)) # second index = first index + num elements in PanelFlux
es

        ArrayFluxes[idx1:idx2] += PanelFluxes  # assign fluxes from one panel to the ith 'bin'

    return ArrayFluxes, S

numpanels = 15
#T_panels = np.ones(numpanels)*T_panel
T_panel = 300

arrayFluxes, S = ArrayFlux(h0, theta, D, T_panel, L, numpanels, X)
```

```
CPU times: user 1.28 s, sys: 4.67 ms, total: 1.28 s
Wall time: 1.29 s
```

In [4]:

```
%%time
# Attempt 3, part 2 -- add if statements for if no transition panels
# Compute view factor for each position along ground
# Left/right were assigned on the assumption that we are viewing the panels so that they point up
to the right

def ViewFactor(h0, theta, D, L, numpanels, X):

    delta_S = X[1]-X[0]

    S = np.arange(0,D*numpanels+L*np.cos(theta),delta_S)
    viewfactors = S*0  # empty mega-array to store view factor values

    # Find critical angles (law of cosines/sines)
    M = np.sqrt(L**2 + D**2 - 2*L*D*np.cos(theta))
    G = np.sqrt(L**2 + D**2 - 2*L*D*np.cos(np.pi-theta))

    leftcrit_angle = np.pi - np.arcsin(L/M * np.sin(theta)) # Make leftcrit obtuse
    rightcrit_angle = np.arcsin(L/G * np.sin(np.pi-theta))

    lefttransitions = S*0
    righttransitions = S*0


    for x in range(0,len(S)):  # for each ground position

        leftedge = np.arctan(h0/S[x])

        panelangles = np.zeros(numpanels)
        topangles = np.zeros(numpanels)
        bottomangles = np.zeros(numpanels)
```

```
        for i in range(1,numpanels+1): # for each panel

            yb = h0
            yt = h0 + L*np.sin(theta)

            xb = D*(i-1)
            xt = D*(i-1) + L*np.cos(theta)

            bottom_angle = np.arctan(yb/(xb-S[x]))
            top_angle = np.arctan(yt/(xt-S[x]))

            # If angle is negative, make it obtuse
            if bottom_angle < 0:
                bottom_angle = bottom_angle + np.pi
            if top_angle < 0:
                top_angle = top_angle + np.pi

            topangles[i-1] = top_angle
            bottomangles[i-1] = bottom_angle

            # Compute panel angle
            if bottom_angle > top_angle:
                panelangles[i-1] = bottom_angle - top_angle
            else:
                panelangles[i-1] = top_angle - bottom_angle

            # Transition Panels:
            # Identify which panel wedge on the left and right of S[x]
            #  intersect with the critical angle lines
            if bottom_angle > leftcrit_angle and top_angle < leftcrit_angle:
                lefttransitions[x] = i
                count = i

                leftedgetop = top_angle # always obtuse

            elif bottom_angle < rightcrit_angle and top_angle > rightcrit_angle:

                righttransitions[x] = i
                rightedge = np.arctan(h0/(D*numpanels - S[x]))

                rightedgetop = top_angle

        # Sum shade wedges of panels that are between left & right transitions
        total_panelangles = 0
        for p in range(int(lefttransitions[x]+1), int(righttransitions[x])):
            total_panelangles += panelangles[p-1]

        if lefttransitions[x]== 0:
            viewfactors[x] = (np.pi - total_panelangles - rightedgetop + rightedge)/np.pi
        elif righttransitions[x]== 0:
            viewfactors[x] = (np.pi - total_panelangles - (np.pi-leftedgetop))/np.pi
        else:
            viewfactors[x] = (np.pi - total_panelangles - (np.pi-leftedgetop) - rightedgetop + ri
ghtedge + leftedge)/np.pi

    return viewfactors, S

viewfactors, S_ = ViewFactor(h0, theta, D, L, numpanels, X)
```

```
CPU times: user 66.8 ms, sys: 1.51 ms, total: 68.3 ms
Wall time: 68 ms

/Users/torin/anaconda3/lib/python3.7/site-packages/ipykernel_launcher.py:25: RuntimeWarning: di
vide by zero encountered in double_scalars
/Users/torin/anaconda3/lib/python3.7/site-packages/ipykernel_launcher.py:39: RuntimeWarning: di
vide by zero encountered in double_scalars
```

In [5]:

```
%%time
# Convert weather data to usable array

count=0
with open('weather_data_data,T,RH,Rs,u.txt','r') as file:
    for line in file:
        count += 1
        if count == 1:
            weird_str = line.split(",")
        if count == 2:
            temps_str = line.split(",")    # air temps
        if count == 3:
            RH_str = line.split(",")       # relative humidity
        if count == 4:
            shortrad_str = line.split(",") # shortwave radiation
        if count == 5:
            wind_str = line.split(",")     # wind speed

# Convert elements in lists from strings to floats
temps = [0]*len(temps_str)
tempsK = [0]*len(temps_str)
for i in range(0,len(temps_str)-1):
    temps[i] = float(temps_str[i])
    tempsK[i] = float(temps_str[i]) +273.
RH = [0]*len(RH_str)
for i in range(0,len(RH_str)-1):
    RH[i] = float(RH_str[i])

shortrad = [0]*len(shortrad_str)
for i in range(0,len(shortrad_str)-1):
    shortrad[i] = float(shortrad_str[i])

wind = [0]*len(wind_str)
for i in range(0,len(wind_str)-1):
    wind[i] = float(wind_str[i])

wind.append(0.2) # arbitrary windspeed so that it's the same size as the rest

print(len(temps_str))

times = np.arange(0,len(temps)*0.25,0.25) # units: hr
plt.rcParams.update({'font.size': 14})

plt.figure(figsize=(18,5.5))
plt.subplot(1,2,1)
plt.plot(times,temps, color='r')
plt.title('Air Temp (ºC)')

plt.subplot(1,2,2)
plt.plot(times,RH, color='blue')
plt.title('Relative Humidity')
plt.show()

plt.figure(figsize=(18,5.5))
plt.subplot(1,2,1)
plt.plot(times,shortrad,color='green')
plt.title('Shortwave Radiation (W/m2)')
plt.xlabel('Time in hours after May 6, 2018, 12am')

plt.subplot(1,2,2)
plt.plot(times,wind, color='orange')
plt.title('Windspeed (m/s)')
plt.xlabel('Time in hours after May 6, 2018, 12am')
plt.show()
```

10945

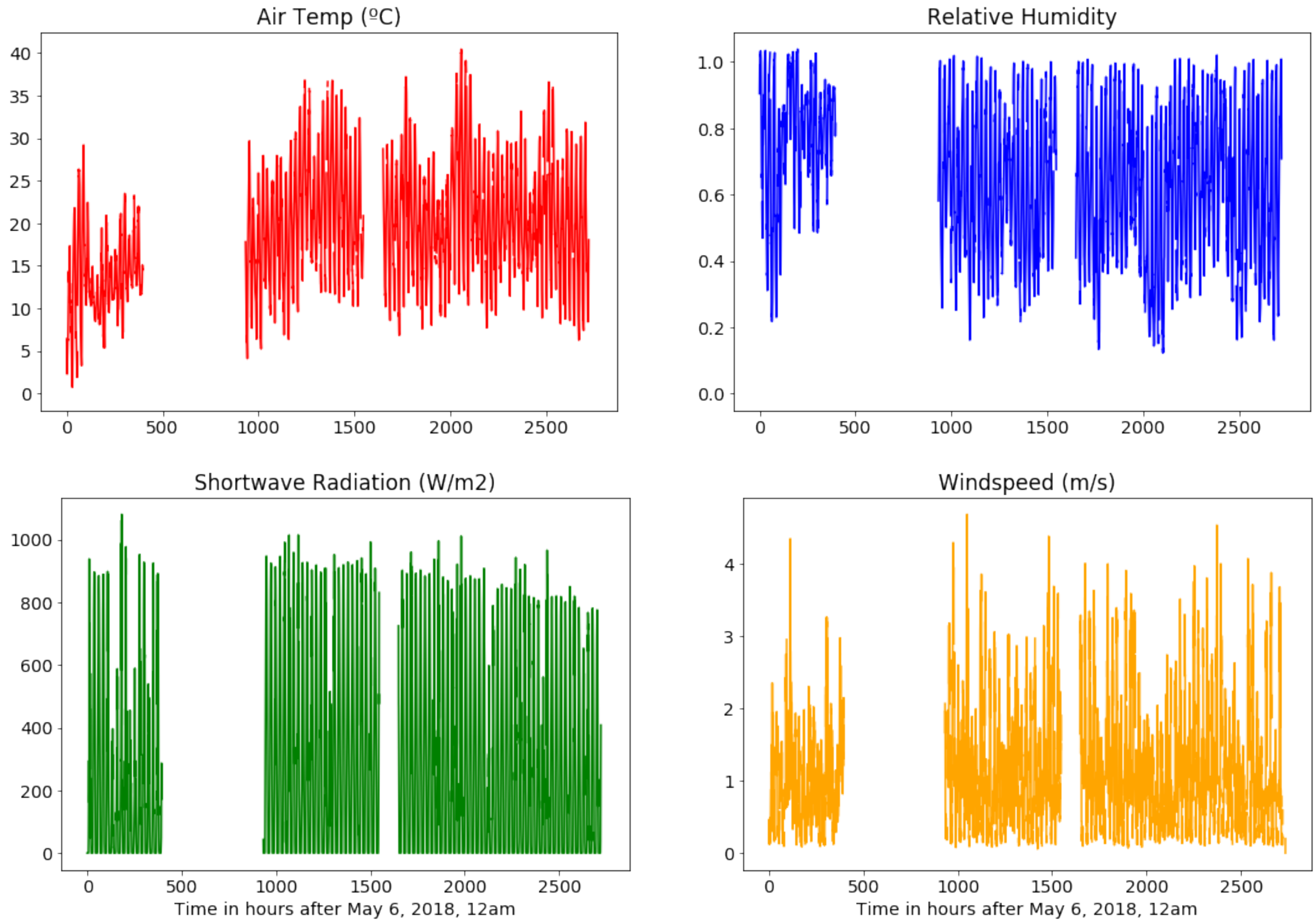


```
CPU times: user 475 ms, sys: 19.7 ms, total: 494 ms
Wall time: 512 ms
```

In [6]:
```
%%time
# Make a time array with 15 min intervals that matches up with the above data
# (should be easy since no breaks in data; breaks are filled with NaNs)
# Compute panel temp at each timestep using the above data

import numpy as np
from scipy import optimize # should I import this inside the def function PanelTemps?

times = np.arange(0,len(temps)*0.25,0.25)

# Input arrays must all be the same length
def PanelTemps(L, airtemp,RH,shortrad,wind,times):

    T_Panel = np.zeros(len(times))
    Lsky = np.zeros(len(times))

    # Energy balance on panel --> get Tp
    sigma = 5.67*10**(-8) # W/m2-K4 ; Stephan Boltzmann constant
    Tref = 298 #K    equation for effieciency only works for Tp ≤ 293
    Eref = 0.135
    effslope = 0.0051 # /K

    for t in range(0,len(times)):
        Tair = airtemp[t] + 273 # K
        Lg = sigma*Tair**4
        Rsun = shortrad[t]
        RH_ = RH[t]
        T_airC = Tair - 273.0

        # Conversions for vapor pressure:
        e_star = 611*np.exp(17.3*(T_airC)/(T_airC+237.3))  # Pa
        e_star = e_star/100  # Pa --> mbar
        e_air = RH_*e_star # vapor pressure of air

        # Longwave radiation from sky for a given Tair:
        Lsky[t] = 1.24*sigma*(e_air/Tair)**(1/7)*Tair**4 # W/m2; where e_air = mbar

        # Convection coefficient
        windspeed = wind[t]
        kair = 0.026 # W/mK ; thermal conductivity of dry air
        visc = 1.57*10**(-5) # m2/s ; kinematic viscosity of air
        Pr = 0.707 # Prandtl number for dry air
        h = 0.036*(kair/L)*(windspeed*L/visc)**(4/5) * Pr**(1/3) # W/(m2-K)


        def Energy_Bal(Tp):
            return (0.8 - Eref*(1-effslope*(Tp-Tref)))*Rsun + Lsky[t] + Lg - 2*sigma*Tp**4 - 2*h*
(Tp-Tair)

        Tp = optimize.root_scalar(Energy_Bal, bracket =[200,600], method='brentq')

        T_Panel[t] = Tp.root
    return T_Panel, Lsky

T_Panel, Lsky = PanelTemps(L, temps, RH, shortrad, wind, times)

# There's no gaps for NaN values, but rather it makes T_panel equal to very small num (1.065e-12
every time)

plt.plot(times[0:100],Lsky[0:100])
plt.xlabel('Time after 12am (hr)')
plt.ylabel('Longwave from Sky (W/m2)')
plt.title('Longwave from Sky for Ground in Full Sun (No Viewfactors)')
plt.show()
```

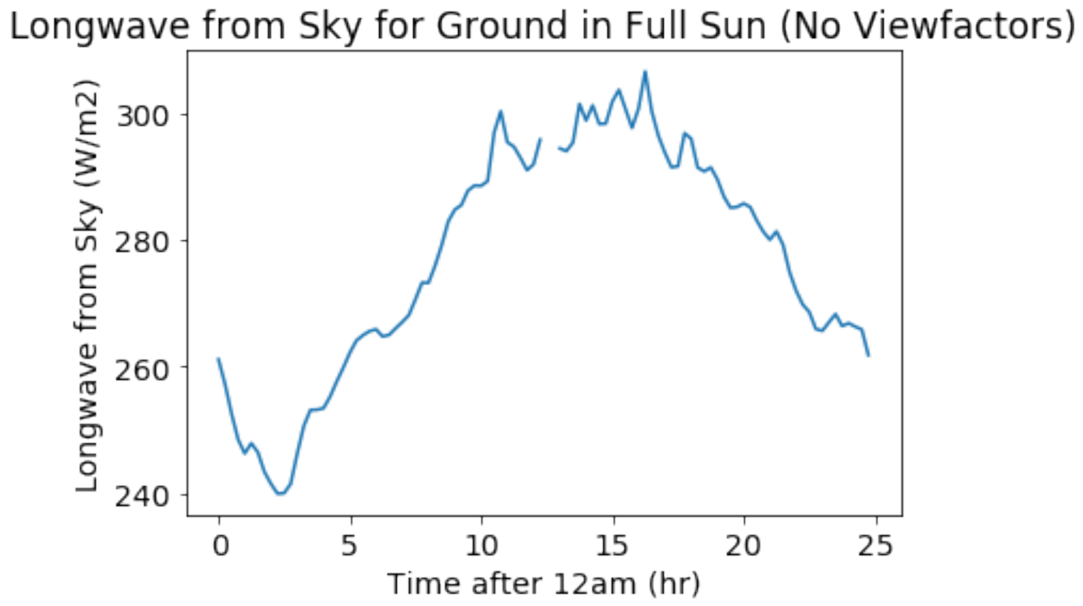


```
CPU times: user 405 ms, sys: 50.3 ms, total: 456 ms
Wall time: 627 ms
```

In [7]:

```
%%time
# Identify all gaps in Tp and linearly interpolate
# ^gaps due to NaNs or optimize didn't find a root? <-- NaNs

def Interpolate(temps):

    temps_adjusted = temps

    for i in range(0,len(temps)):
        if temps[i] < 1:
            if temps[i-1] > 1:
            # Identify point just before NaN section
                x1 = i-1
                y1 = temps[i-1]
                #print(x1,y1)
            if temps[i+1] > 1:
            # Identify point just after NaN section
                x2 = i+1
                y2 = temps[i+1]
                #print(x2,y2)

                xp = np.array([x1,x2])
                yp = np.array([y1,y2])
                #print('xp = {}'.format(xp))
                #print('yp = {}'.format(yp))
                idx1 = x1+1
                idx2 = x2

                to_be_interp = np.arange(idx1,idx2) #temps[idx1:idx2]
                temps_interp = np.interp(to_be_interp,xp,yp)
                temps_adjusted[idx1:idx2] = temps_interp
                #print(temps_adjusted)
    return temps_adjusted

#testtemps = np.array([4,6,0.1,0.1,12,14,16,18,0.1,22,24])
#testtemps_adjusted = Interpolate(testtemps)
#print(testtemps_adjusted)

T_panel_adjusted = Interpolate(T_Panel)

plt.figure(figsize=(9,6))
plt.plot(times[0:100],T_panel_adjusted[0:100],label='Panel Temp')
plt.plot(times[0:100],tempsK[0:100],label='Air Temp')
plt.xlabel('Time after 12am (hr)')
plt.ylabel('Temperature (K)')
plt.ylim([270,310])
plt.legend()
plt.show()

plt.plot(times,T_panel_adjusted)

print(T_panel_adjusted[6590:6600])

# Indices of Tpanel...
# first chunk: 0-1583
# second chunk: 3732 - 6186
# third chunk: 6597 - end
```

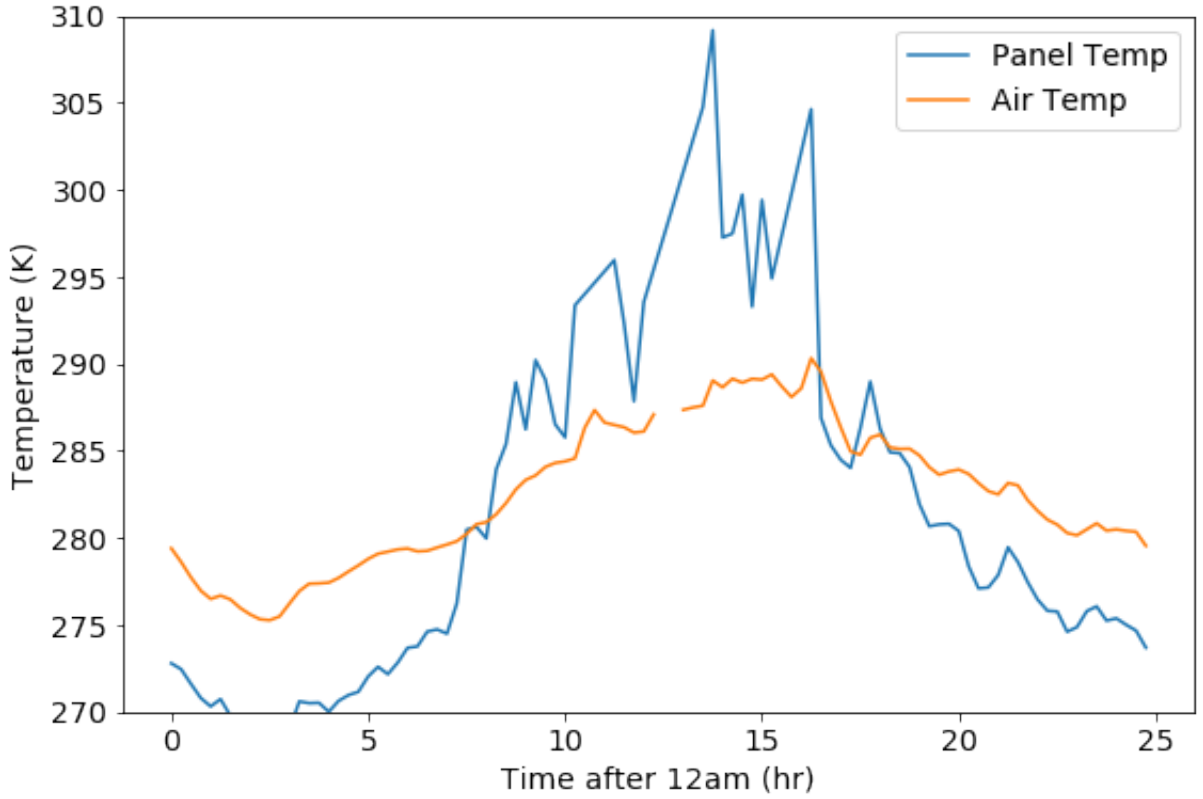


```
[316.75294702 316.76253186 316.77211669 316.78170153 316.79128636
 316.8008712  316.81045603 308.70711143 311.86768775 307.09371395]
CPU times: user 130 ms, sys: 5.87 ms, total: 136 ms
Wall time: 135 ms
```

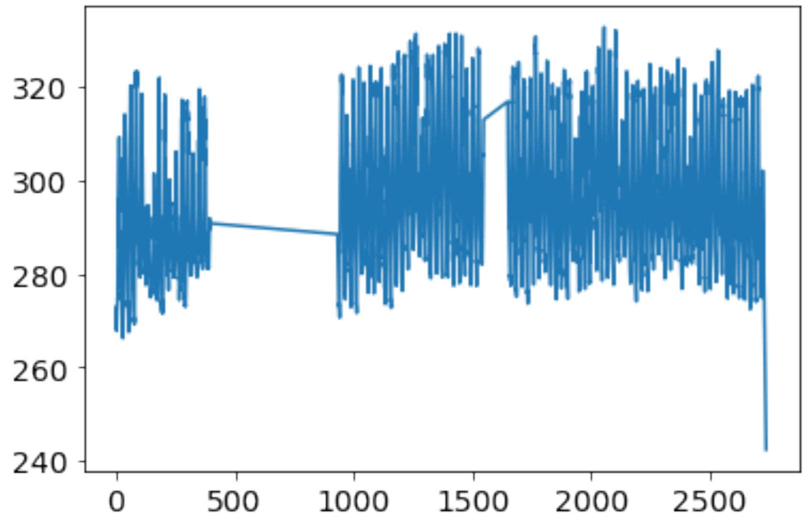

In [8]:

```
%%time
# Multiply Lsky vector by Viewfactor vector (can't do this directly because num of times ≠ num of spaces)
# S_ = space elements in VF function; only includes non-zero flux spaces (omits flat sections)

Lsky_received = np.zeros([len(times),len(S_)])
# across a row = across space
# down a column = across time

for t in range(0,len(Lsky)):
    Lsky_received[t,:] = Lsky[t]*viewfactors

print(Lsky_received)
```

```
[[163.74821191 156.89492482 150.24943157 ... 252.9382214  252.9382214
  252.9382214 ]
 [161.30120589 154.55033234 148.00414742 ... 249.15838563 249.15838563
  249.15838563]
 [158.35096796 151.72356952 145.2971159  ... 244.60121871 244.60121871
  244.60121871]
 ...
 [         nan          nan          nan ...          nan          nan
           nan]
 [         nan          nan          nan ...          nan          nan
           nan]
 [  0.           0.           0.         ...   0.           0.
    0.        ]]
CPU times: user 35.1 ms, sys: 15.5 ms, total: 50.6 ms
Wall time: 50.2 ms
```

In [9]:

```
%%time
# Plot maximum radiation from panels integrated over time for each time chunk

maxflux1 = np.empty([1580,len(S_)])
maxflux2 = np.empty([2452,len(S_)])
maxflux3 = np.empty([4348,len(S_)])

for x in range(0,len(S_)):
    for t in range (0,1580):
        maxflux1[t,x] = sigma*T_panel_adjusted[t]**4
    for t in range (3732,6184):
        idx = t-3732
        maxflux2[idx,x] = sigma*T_panel_adjusted[t]**4
    for t in range (6597,10945):
        idx = t-6597
        maxflux3[idx,x] = sigma*T_panel_adjusted[t]**4

maxflux1_sum = np.nansum(maxflux1, axis=0)*900
maxflux2_sum = np.nansum(maxflux2, axis=0)*900
maxflux3_sum = np.nansum(maxflux3, axis=0)*900
```

```
CPU times: user 3.73 s, sys: 84.2 ms, total: 3.81 s
Wall time: 3.67 s
```

In [10]:
```
%%time

plt.plot(S_,maxflux1_sum,'-')
plt.plot(S_,maxflux2_sum,'-')
plt.plot(S_,maxflux3_sum,'-')
```

```
CPU times: user 11.3 ms, sys: 1.33 ms, total: 12.6 ms
Wall time: 11.6 ms
```

Out[10]: [<matplotlib.lines.Line2D at 0x7fb1c04c8860>]

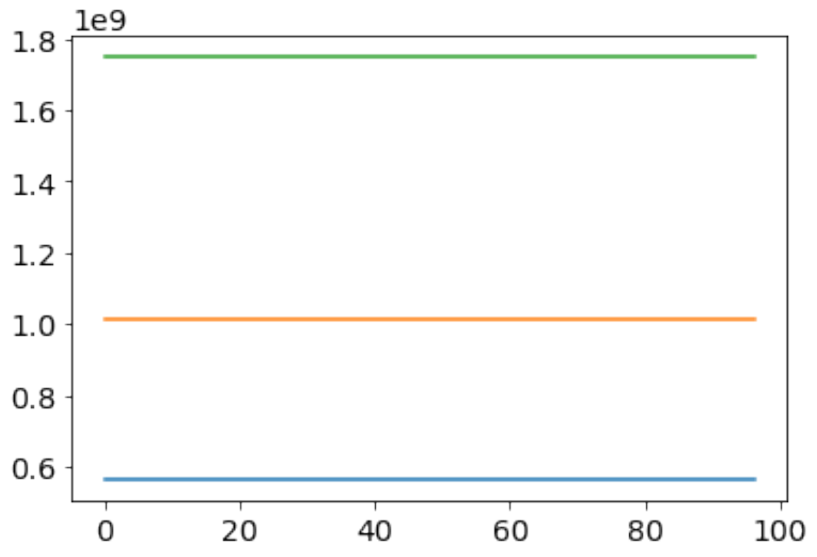

In [11]:
```
%%time

print('Max Flux Sum 1 is {0:1.3E}'.format(np.amax(maxflux1_sum)))
print()
print('Max Flux Sum 1 is {0:1.3E}'.format(np.amax(maxflux2_sum)))
print()
print('Max Flux Sum 1 is {0:1.3E}'.format(np.amax(maxflux3_sum)))
```

```
Max Flux Sum 1 is 5.642E+08

Max Flux Sum 1 is 1.011E+09

Max Flux Sum 1 is 1.748E+09
CPU times: user 256 µs, sys: 102 µs, total: 358 µs
Wall time: 301 µs
```

In [12]:
```
%%time
# Create 3D pcolormesh() plot that displays flux vs space vs time (FOR FIRST CHUNK)
# spaces = x axis, times = y axis
# 0:1583

dt = 0.25 # hr (15 min)
ds = X[1]-X[0]

S_end = D*numpanels + L*np.cos(theta)

timegrid, spacegrid = np.mgrid[slice(0, 395, dt),
                               slice(0, S_end, ds)]
# Above: 395 hour timesteps bc 395*4 = 1580 = number of time elements in first chunk

allArrayFluxes = np.empty([1580,len(S_)])

# Number of points in S_ with "resolution" 1000 = 225
# First index in S: X[0]/(step size) = (212+1.42)/0.4255 = 501
# (aka S[501] = S_[0])
# Second index in S: first index + number of points in S_ + 1 because end not inclusive = 501 + 2
25 +1 = 727

# Number of points in S_ with "resolution" 4000 = 900
# First index in S: X[0]/(new delta_S) = (212+1.42)/0.1064 = 2004
# Second index in S: first index + number of points in S_ + 1= 2004 + 900 + 1 = 2905

# 12am May 6 - 11am May 22
print('FLUXES  FOR  THE  FIRST 16 DAYS (t=0 @ 6 May 12am)')
# Below: for each time, input paneltemp into flux function & assign all 900 nonzero fluxes to tim
e-space array
for t in range(0,1580):
    paneltemp = T_panel_adjusted[t]
    arrayFluxes, S = ArrayFlux(h0, theta, D, paneltemp, L, numpanels, X)
    allArrayFluxes[t,:] = arrayFluxes[2004:2906]

    # [2004:2906] is the nonzero section; I did this to be consistent with S_ , viewfactors, and
Lsky_received

longwavetotal = Lsky_received[0:1580,:] + allArrayFluxes[0:1580,:]
```

```
FLUXES  FOR  THE  FIRST 16 DAYS (t=0 @ 6 May 12am)
CPU times: user 33min 41s, sys: 5.7 s, total: 33min 47s
Wall time: 33min 56s
```

In [13]:

```
%%time
# Create "pinstriped" pcolormesh() plot for flux in control plot (w/o solar panels, aka only Lsky
)
# spaces = x axis, times = y axis
# 0:1583

dt = 0.25 # hr (15 min)
ds = X[1]-X[0]

S_end = D*numpanels + L*np.cos(theta)

timegrid, spacegrid = np.mgrid[slice(0, 395, dt),
                               slice(0, S_end, ds)]

longwavetotal_nopanels = np.empty([1580,len(S_)])

for col in range(0,len(S_)):
    longwavetotal_nopanels[:,col] = Lsky[0:1580]

plt.figure(figsize=(18,6))
plt.subplot(122)
plt.pcolormesh(timegrid, spacegrid, longwavetotal_nopanels)
plt.xlabel('Time (hr) after 12am May 6, 2015')
plt.ylabel('Position (m)')
plt.colorbar(label='Heat Flux (W/m2)')
plt.clim(200,1405)
plt.title('Heat Flux at Ground from Sky (Control Plot)')

plt.subplot(121)
plt.pcolormesh(timegrid, spacegrid, longwavetotal)
plt.xlabel('Time (hr) after 12am May 6, 2015')
plt.ylabel('Position (m)')
plt.colorbar(label='Heat Flux (W/m2)')
plt.title('Heat Flux at Ground from Panels & Sky')
plt.show()
```

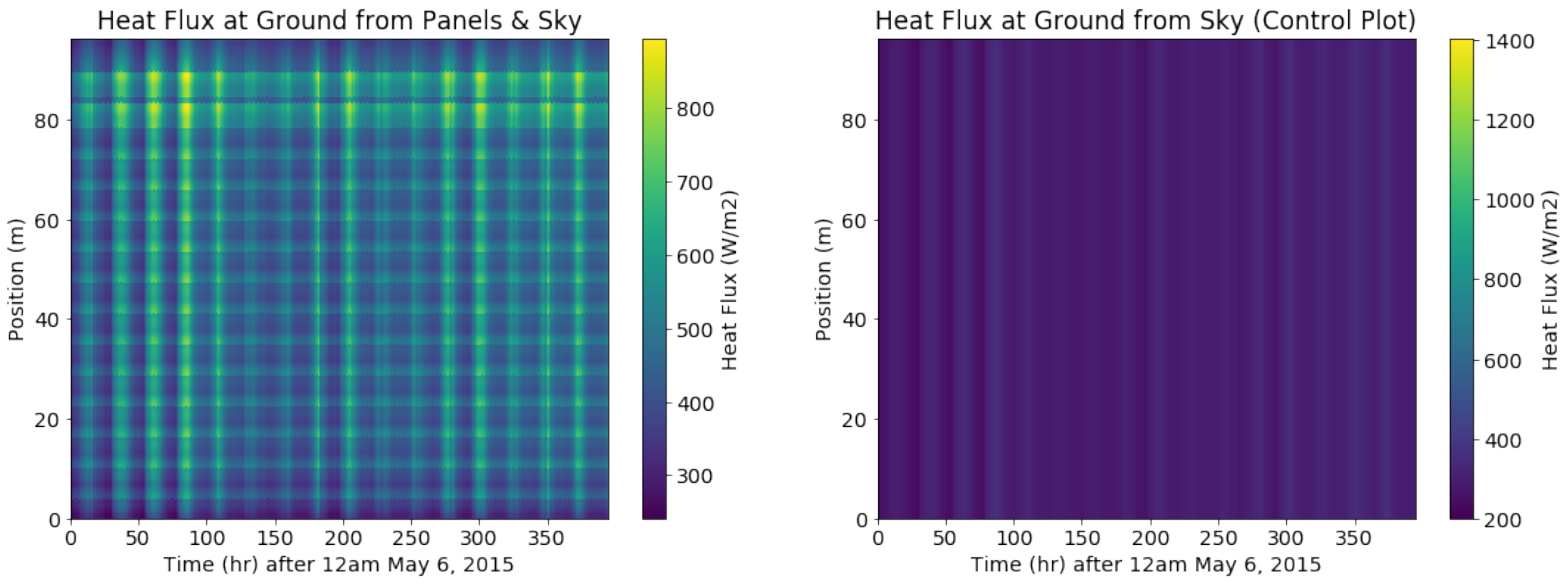


```
CPU times: user 5.96 s, sys: 173 ms, total: 6.13 s
Wall time: 2.03 s
```

In [31]:

```
%%time
# Make plots of longwave vs. position for...
# Only panels (already plotted earlier)
# Only sky (vf but no panel rad)
# sky + panels
# no panels (flat line)

plt.figure(figsize=(12,6))

Lsky_t0 = Lsky_received[0,:]
Lsky_tot_t0 = np.ones(np.shape(S_))*261.1
```

```python
plt.plot(S_, Lsky_t0, label='Lsky total = 261 W/(m2)')
plt.plot(S[0:3000],arrayFluxes[0:3000], label='From Panels')
plt.plot(S_,Lsky_tot_t0, label='Lsky total (full sun)')
plt.ylim([70,450])
plt.xlim([-5,100])

for i in range(0,numpanels):
    x1 = D*i
    x2 = D*i + L*np.cos(theta)
    # Shade region under panels
    plt.axvspan(x1,x2,color='gainsboro')

for i in range(numpanels-1,numpanels):
    plt.axvspan(x1,x2,color='gainsboro',label='Region Under Panels')

print(Lsky[0])
print('Longwave Received from Sky + Panels at t=0')
plt.xlabel('Ground position (m)')
plt.ylabel('Heat Flux (W/m2)')
plt.rcParams.update({'font.size': 12})
plt.legend(loc='center')
plt.show()

# Plot same thing as above, but only effect from panels, and change ylim/xlim to zoom in
print('Longwave Received from Sky + Panels at t=0')
plt.figure(figsize=(15,4))
plt.plot(S[0:3000],arrayFluxes[0:3000], label='From Panels', color='darkorange')
plt.ylim([70,450])
plt.xlim([0,30])
plt.xlabel('Ground position (m)')
plt.ylabel('Heat Flux (W/m2)')

for i in range(0,numpanels):
    x1 = D*i
    x2 = D*i + L*np.cos(theta)
    # Shade region under panels
    plt.axvspan(x1,x2,color='gainsboro')

for i in range(numpanels-1,numpanels):
    plt.axvspan(x1,x2,color='gainsboro',label='Region Under Panels')

#plt.legend(loc='upper left')
plt.show()

plt.figure(figsize=(15,5))
plt.plot(S_,longwavetotal[1579,:],label='Solar Array')
flatline = Lsky[1579]*np.ones(len(S_))
plt.plot(S_,flatline,label='Control Area',color='green')
plt.ylim([300,700])
plt.xlabel('Ground position (m)')
plt.ylabel('Heat Flux (W/m2)')
plt.legend()

for i in range(0,numpanels):
    x1 = D*i
    x2 = D*i + L*np.cos(theta)
    # Shade region under panels
    plt.axvspan(x1,x2,color='gainsboro')

for i in range(numpanels-1,numpanels):
    plt.axvspan(x1,x2,color='gainsboro',label='Region Under Panels')
```

261.0834623027543
Longwave Received from Sky + Panels at t=0

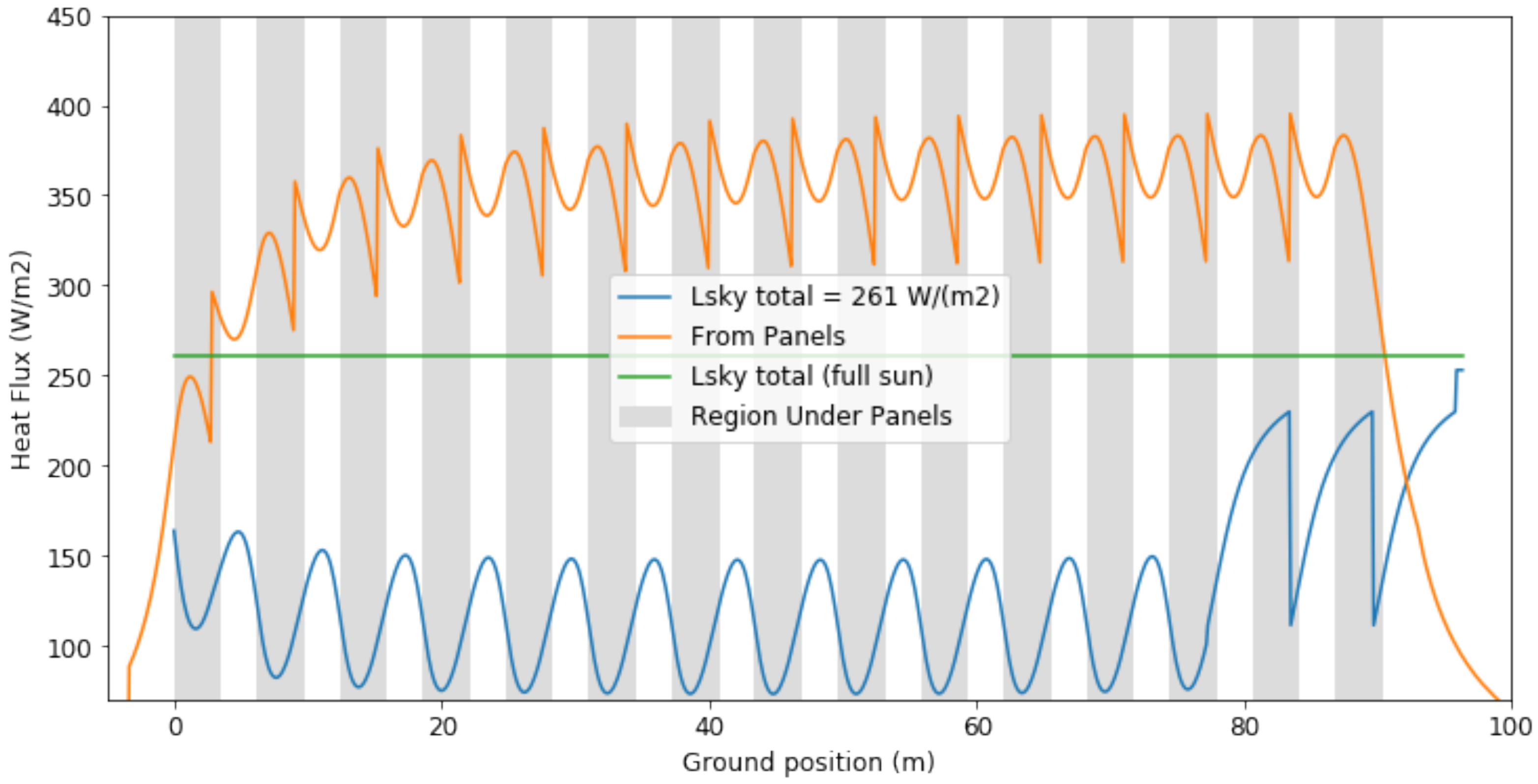

Longwave Received from Sky + Panels at t=0

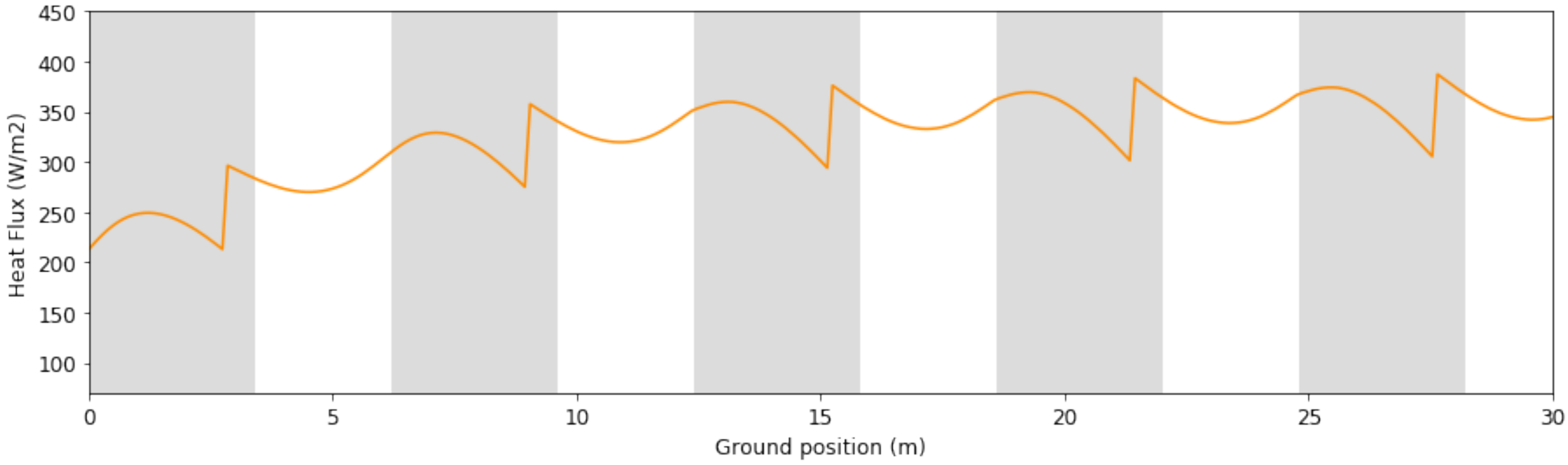

CPU times: user 283 ms, sys: 11.3 ms, total: 295 ms
Wall time: 294 ms

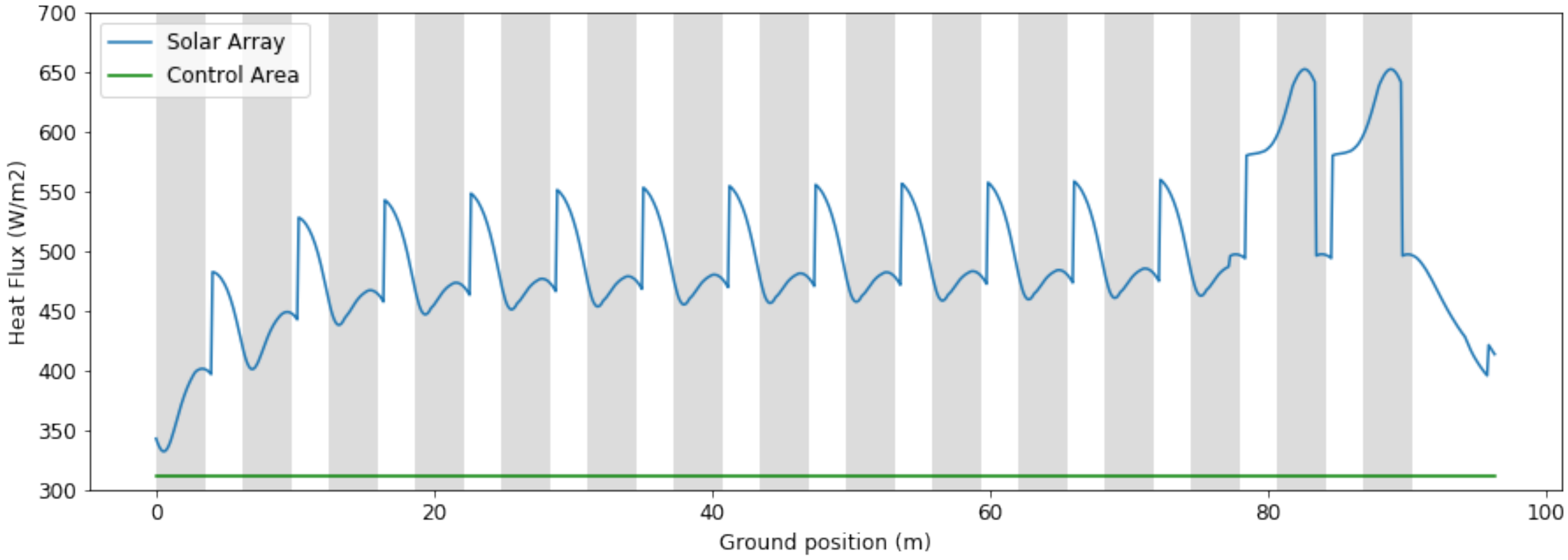

In [32]:
```
%%time
# flux vs space vs time (FOR SECOND CHUNK)
# spaces = x axis, times = y axis
# 3732:6186

timegrid2, spacegrid2 = np.mgrid[slice(0, 613, dt),
                                 slice(0, S_end, ds)]
# Above: 613 hour timesteps bc 613*4 = 2452 = (6186-3732) rounded to nearest multiple of 4

allArrayFluxes2 = np.empty([2452,len(S_)])

# 3732 = 38.875 days after May 6 = 9pm June 13
# 6184 = 64.417 days after May 6 = 10am July 9
print('Fluxes from 9pm June 13 to 10am July 9, 2015')

for t in range(0,2452):
    idx = t + 3732
    paneltemp = T_panel_adjusted[idx]
    arrayFluxes, S = ArrayFlux(h0, theta, D, paneltemp, L, numpanels, X)
    allArrayFluxes2[t,:] = arrayFluxes[2004:2906]

longwavetotal2 = Lsky_received[3732:6184,:] + allArrayFluxes2[:,:]

print(np.shape(longwavetotal2))
```

```
Fluxes from 9pm June 13 to 10am July 9, 2015
(2452, 902)
CPU times: user 52min 16s, sys: 8.13 s, total: 52min 25s
Wall time: 52min 38s
```

In [33]:
```
%%time
# flux vs space vs time (FOR THIRD CHUNK)
# spaces = x axis, times = y axis
# 6597:10945 (end)

timegrid3, spacegrid3 = np.mgrid[slice(0, 1087, dt),
                                 slice(0, S_end, ds)]
# Above: 1087 hour timesteps bc 1087*4 = (10945-6597)

allArrayFluxes3 = np.empty([4348,len(S_)])

print('Fluxes from 5pm July 13 to 12am Aug 28, 2015')

for t in range(0,4348):
    idx = t + 6597
    paneltemp = T_panel_adjusted[idx]
    arrayFluxes, S = ArrayFlux(h0, theta, D, paneltemp, L, numpanels, X)
    allArrayFluxes3[t,:] = arrayFluxes[2004:2906]

longwavetotal3 = Lsky_received[6597:,:] + allArrayFluxes3[:,:]

print(np.shape(longwavetotal3))
```

```
Fluxes from 5pm July 13 to 12am Aug 28, 2015
(4348, 902)
CPU times: user 1h 33min 8s, sys: 15.5 s, total: 1h 33min 24s
Wall time: 1h 33min 51s
```

In [34]:
```
%%time
# Create pcolormesh() plot for all three time chunks

timegrid, spacegrid = np.mgrid[slice(0, 395, dt),
                               slice(0, S_end, ds)]
plt.figure(figsize=(20,6))
plt.subplot(131)
plt.pcolormesh(timegrid, spacegrid, longwavetotal)
plt.xlabel('Time (hr) after 12am May 6, 2015')
plt.ylabel('Position (m)')
plt.colorbar(label='Heat Flux (W/m2)')
plt.clim(200,825)
plt.title('Heat Flux at Ground from Panels & Sky')

plt.subplot(132)
plt.pcolormesh(timegrid2, spacegrid2, longwavetotal2)
plt.xlabel('Time (hr) after 19pm June 13, 2015')
plt.ylabel('Position (m)')
plt.colorbar(label='Heat Flux (W/m2)')
plt.clim(200,825)
plt.title('Heat Flux at Ground from Panels & Sky')

plt.subplot(133)
plt.pcolormesh(timegrid3, spacegrid3, longwavetotal3)
plt.xlabel('Time (hr) after 5pm July 13, 2015')
plt.ylabel('Position (m)')
plt.colorbar(label='Heat Flux (W/m2)')
plt.clim(200,825)
plt.title('Heat Flux at Ground from Panels & Sky')
plt.show()
```

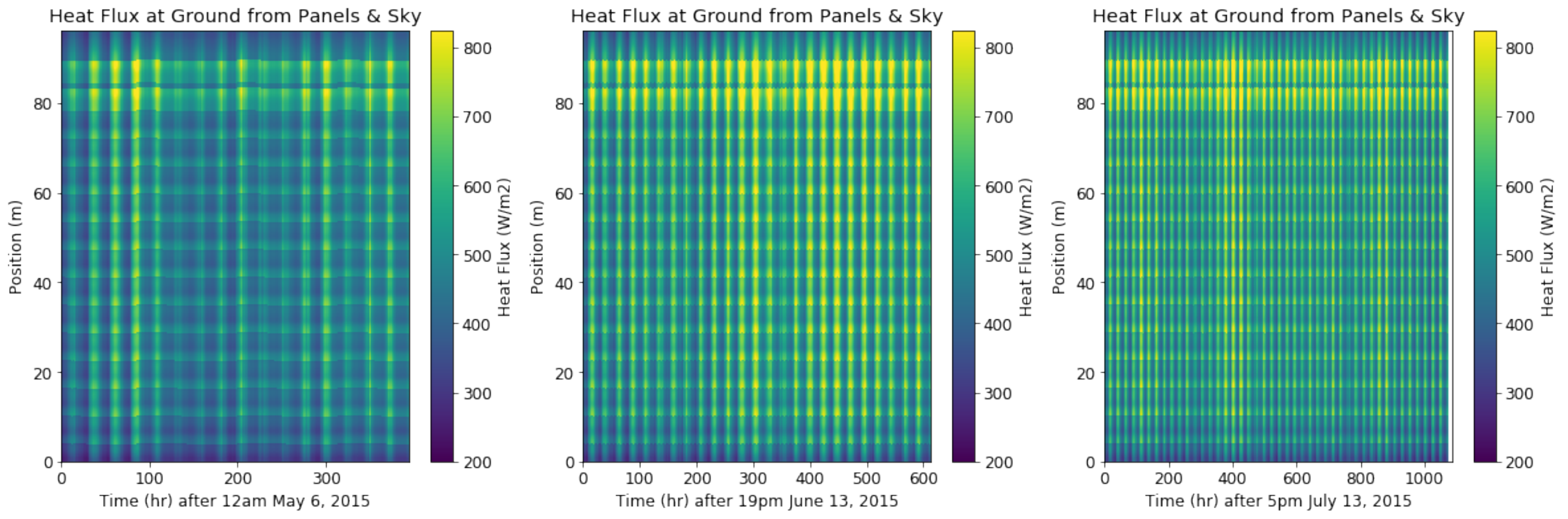


```
CPU times: user 11.6 s, sys: 376 ms, total: 11.9 s
Wall time: 4.85 s
```

In [35]:

```
%%time
# Each 'fluxsum' is a 1D array across space (summed over time)
# 900 sec per 15 min -> J/s-m2 * 900s/15min = J/m2

fluxsum1 = np.nansum(longwavetotal, axis=0)*900  # 12am May 6 - 11am May 22
fluxsum2 = np.nansum(longwavetotal2, axis=0)*900 # 9pm June 13 - 10am July 9
fluxsum3 = np.nansum(longwavetotal3, axis=0)*900 # 5pm July 13 - 12am Aug 28

# longwave total, no panels
lwtot1_np = np.empty([1580,len(S_)])
lwtot2_np = np.empty([2452,len(S_)])
lwtot3_np = np.empty([4348,len(S_)])

for col in range(0,len(S_)):
    lwtot1_np[:,col] = Lsky[0:1580]
    lwtot2_np[:,col] = Lsky[3732:6184]
    lwtot3_np[:,col] = Lsky[6597:]

lwtot1_npsum = np.nansum(lwtot1_np, axis=0)*900
lwtot2_npsum = np.nansum(lwtot2_np, axis=0)*900
lwtot3_npsum = np.nansum(lwtot3_np, axis=0)*900

anomaly1 = fluxsum1-lwtot1_npsum
anomaly2 = fluxsum2-lwtot2_npsum
anomaly3 = fluxsum3-lwtot3_npsum

# Plot maximum radiation from panels integrated over time for each time chunk

maxflux1 = np.empty([1580,len(S_)])
maxflux2 = np.empty([2452,len(S_)])
maxflux3 = np.empty([4348,len(S_)])

for x in range(0,len(S_)):
    for t in range (0,1580):
        maxflux1[t,x] = sigma*T_panel_adjusted[t]**4
    for t in range (3732,6184):
        idx = t-3732
        maxflux2[idx,x] = sigma*T_panel_adjusted[t]**4
    for t in range (6597,10945):
        idx = t-6597
        maxflux3[idx,x] = sigma*T_panel_adjusted[t]**4

maxflux1_sum = np.nansum(maxflux1, axis=0)*900
maxflux2_sum = np.nansum(maxflux2, axis=0)*900
maxflux3_sum = np.nansum(maxflux3, axis=0)*900
```

```
CPU times: user 5.38 s, sys: 120 ms, total: 5.5 s
Wall time: 3.81 s
```

In [44]:
```
%%time

# Plot difference in evaporation potential

Hvap = 44 # kJ/mol; Latent heat of vaporization of water
rho = 1000 # kg/m3; density of water
MW = 0.018 # kg/mol; molar mass of water

# multiply by 100 to go from m -> cm, divide by 1000 to go from J -> kJ

evap_anomaly1 = anomaly1*MW/(rho*Hvap)*100/1000
evap_anomaly2 = anomaly2*MW/(rho*Hvap)*100/1000
evap_anomaly3 = anomaly3*MW/(rho*Hvap)*100/1000

plt.figure(figsize=(17,4))
plt.plot(S_,evap_anomaly3, label='7/13-8/28', color='green')
plt.plot(S_,evap_anomaly2, label='6/13-7/9', color='dodgerblue')
plt.plot(S_,evap_anomaly1, label='5/6-5/22', color='r')


# Below: horizontal max flux lines if all the ground saw was panels (sanity check)
# do not include in final graph
#plt.plot(S_,maxflux1_sum,'-', color='r')
#plt.plot(S_,maxflux2_sum,'-', color='deepskyblue')
#plt.plot(S_,maxflux3_sum,'-', color='lawngreen')

plt.legend()
plt.xlabel('Position (m)')
plt.ylabel('Difference in Evaporation Potential (cm)')
#plt.title('Additional evaporation from ground under panels vs full sun')

for i in range(0,numpanels):
    x1 = D*i
    x2 = D*i + L*np.cos(theta)
    # Shade region under panels
    plt.axvspan(x1,x2,color='gainsboro')

for i in range(numpanels-1,numpanels):
    plt.axvspan(x1,x2,color='gainsboro',label='Region Under Panels')

plt.show()
```

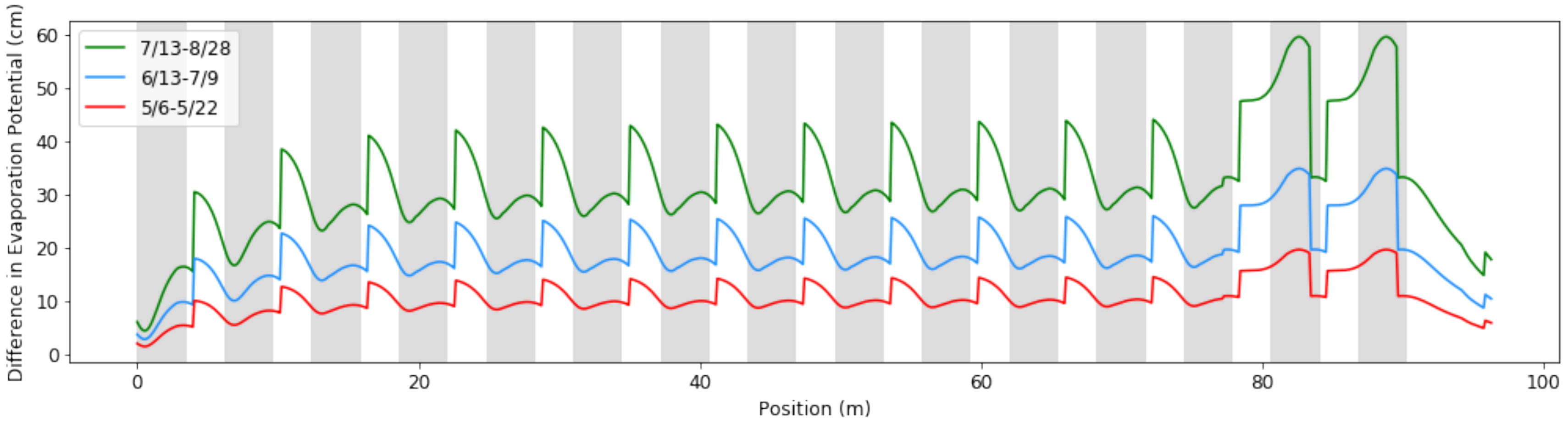


```
CPU times: user 163 ms, sys: 4.88 ms, total: 168 ms
Wall time: 168 ms
```

In [45]:
```
%%time

def SaveArray(data, c, filename='output.txt'):
    teststr = np.array2string(data, separator=',', max_line_width=None)
    if c == 'overwrite':
        testfile = open(filename, 'w')
    if c == 'append':
        testfile = open(filename, 'a')
    testfile.write(teststr)
    testfile.write(',')
    testfile.close()

def LineBreak(c, filename='output.txt'):
    if c == 'overwrite':
        testfile = open(filename, 'w')
    if c == 'append':
        testfile = open(filename, 'a')
    testfile.write('\n')
    testfile.close()
```

```
CPU times: user 3 µs, sys: 0 ns, total: 3 µs
Wall time: 5.25 µs
```

In [53]:
```
%%time

print('Energy Maximums')
print()
print('May 6 to May 22 Max is {0:1.3E} J/m2 at position {1:2.5}m'.format(np.amax(anomaly1),S_[np.
argmax(anomaly1)]))
print()
print('June 13 to July 9 Max is {0:1.3E} J/m2 at position {1:2.5}m'.format(np.amax(anomaly2),S_[n
p.argmax(anomaly2)]))
print()
print('July 13 to August 28 Max is {0:1.3E} J/m2 at position {1:2.5}m'.format(np.amax(anomaly3),S
_[np.argmax(anomaly3)]))
print()
print('Evaporation Potential Maximums')
print()
print('May 6 to May 22 Max is {0:.2f} cm at position {1:2.5}m'.format(np.amax(evap_anomaly1),S_[n
p.argmax(evap_anomaly1)]))
print()
print('June 13 to July 9 Max is {0:.2f} cm at position {1:2.5}m'.format(np.amax(evap_anomaly2),S_
[np.argmax(evap_anomaly2)]))
print()
print('July 13 to August 28 Max is {0:.2f} cm at position {1:2.5}m'.format(np.amax(evap_anomaly3)
,S_[np.argmax(evap_anomaly3)]))
print()
```

```
Energy Maximums

May 6 to May 22 Max is 4.790E+08 J/m2 at position 88.812m

June 13 to July 9 Max is 8.511E+08 J/m2 at position 88.812m

July 13 to August 28 Max is 1.456E+09 J/m2 at position 88.812m

Evaporation Potential Maximums

May 6 to May 22 Max is 19.60 cm at position 88.812m

June 13 to July 9 Max is 34.82 cm at position 88.812m

July 13 to August 28 Max is 59.58 cm at position 88.812m

CPU times: user 487 µs, sys: 485 µs, total: 972 µs
Wall time: 517 µs
```

In [56]:

```
%%time

anomalyfilename1 = 'anomaly1.txt'
LineBreak(c='overwrite', filename=anomalyfilename1)
SaveArray(data=anomaly1, c='append', filename=anomalyfilename1)

anomalyfilename2 = 'anomaly2.txt'
LineBreak(c='overwrite', filename=anomalyfilename2)
SaveArray(data=anomaly2, c='append', filename=anomalyfilename2)

anomalyfilename3 = 'anomaly3.txt'
LineBreak(c='overwrite', filename=anomalyfilename3)
SaveArray(data=anomaly3, c='append', filename=anomalyfilename3)

evap_anomalyfilename1 = 'evap_anomaly1.txt'
LineBreak(c='overwrite', filename=evap_anomalyfilename1)
SaveArray(data=evap_anomaly1, c='append', filename=evap_anomalyfilename1)

evap_anomalyfilename2 = 'evap_anomaly2.txt'
LineBreak(c='overwrite', filename=evap_anomalyfilename2)
SaveArray(data=evap_anomaly2, c='append', filename=evap_anomalyfilename2)

evap_anomalyfilename3 = 'evap_anomaly3.txt'
LineBreak(c='overwrite', filename=evap_anomalyfilename3)
SaveArray(data=evap_anomaly3, c='append', filename=evap_anomalyfilename3)
```

```
CPU times: user 29.2 ms, sys: 2.63 ms, total: 31.8 ms
Wall time: 31.3 ms
```

In [59]:

```
%%time
# Break anomaly arrays into smaller chunks to identify local max/mins
# 1) Identify position of two arbitrary points before and after each local max/min
# 2) Divide that position by delta_S, 0.4255 m, to get bounds for anomaly_1_x
# Format: anomaly[timechunk]_panel
# e.g. anomaly1_2 corresponds to 1st time chunk and max/min close to 2nd panel

anomaly1_2 = anomaly1[72:140]
Smax1_2 = np.argmax(anomaly1_2) + 72 # add 72 to adjust to absolute position
Smin1_2 = np.argmin(anomaly1_2) + 72
print('Energy Maximums')
print()
print('May 6 to May 22, 2nd panel Max is {0:1.3E} J/m2 at position {1:2.5}m'.format(np.amax(anoma
ly1_2),S_[Smax1_2]))
print('May 6 to May 22, 2nd panel Min is {0:1.3E} J/m2 at position {1:2.5}m'.format(np.amin(anoma
ly1_2),S_[Smin1_2]))
print()
# Now that 2nd panel max position known, can use as index for 2nd & 3rd time chunks
print('June 13 to July 9, 2nd panel Max is {0:1.3E} J/m2 at position {1:2.5}m'.format(anomaly2[Sm
ax1_2],S_[Smax1_2]))
print('June 13 to July 9, 2nd panel Min is {0:1.3E} J/m2 at position {1:2.5}m'.format(anomaly2[Sm
in1_2],S_[Smin1_2]))
print()
print('July 13 to August 28, 2nd panel Max is {0:1.3E} J/m2 at position {1:2.5}m'.format(anomaly3
[Smax1_2],S_[Smax1_2]))
print('July 13 to August 28, 2nd panel Min is {0:1.3E} J/m2 at position {1:2.5}m'.format(anomaly3
[Smin1_2],S_[Smin1_2]))
print()

evap_anomaly1_2 = evap_anomaly1[72:140]
evap_Smax1_2 = np.argmax(evap_anomaly1_2) + 72 # add 72 to adjust to absolute position
evap_Smin1_2 = np.argmin(evap_anomaly1_2) + 72
print('Evaporation Potential Maximums')
print()
print('May 6 to May 22, 2nd panel Max is {0:.2f} cm at position {1:2.5}m'.format(np.amax(evap_ano
maly1_2),S_[evap_Smax1_2]))
print('May 6 to May 22, 2nd panel Min is {0:.2f} cm at position {1:2.5}m'.format(np.amin(evap_ano
maly1_2),S_[evap_Smin1_2]))
print()
# Now that 2nd panel max position known, can use as index for 2nd & 3rd time chunks
print('June 13 to July 9, 2nd panel Max is {0:.2f} cm at position {1:2.5}m'.format(evap_anomaly2[
Smax1_2],S_[evap_Smax1_2]))
print('June 13 to July 9, 2nd panel Min is {0:.2f} cm at position {1:2.5}m'.format(evap_anomaly2[
Smin1_2],S_[evap_Smin1_2]))
print()
print('July 13 to August 28, 2nd panel Max is {0:.2f} cm at position {1:2.5}m'.format(evap_anomal
y3[Smax1_2],S_[evap_Smax1_2]))
print('July 13 to August 28, 2nd panel Min is {0:.2f} cm at position {1:2.5}m'.format(evap_anomal
y3[Smin1_2],S_[evap_Smin1_2]))
print()
```

```
Energy Maximums

May 6 to May 22, 2nd panel Max is 3.086E+08 J/m2 at position 10.26m
May 6 to May 22, 2nd panel Min is 1.567E+08 J/m2 at position 7.6949m

June 13 to July 9, 2nd panel Max is 5.533E+08 J/m2 at position 10.26m
June 13 to July 9, 2nd panel Min is 2.873E+08 J/m2 at position 7.6949m

July 13 to August 28, 2nd panel Max is 9.399E+08 J/m2 at position 10.26m
July 13 to August 28, 2nd panel Min is 4.795E+08 J/m2 at position 7.6949m

Evaporation Potential Maximums

May 6 to May 22, 2nd panel Max is 12.62 cm at position 10.26m
May 6 to May 22, 2nd panel Min is 6.41 cm at position 7.6949m

June 13 to July 9, 2nd panel Max is 22.63 cm at position 10.26m
June 13 to July 9, 2nd panel Min is 11.75 cm at position 7.6949m

July 13 to August 28, 2nd panel Max is 38.45 cm at position 10.26m
July 13 to August 28, 2nd panel Min is 19.62 cm at position 7.6949m

CPU times: user 535 µs, sys: 468 µs, total: 1 ms
Wall time: 619 µs
```

In [63]:

```
%%time

anomaly1_1 = anomaly1[0:71]
Smax1_1 = np.argmax(anomaly1_1)
Smin1_1 = np.argmin(anomaly1_1)

anomaly1_2 = anomaly1[72:140]
Smax1_2 = np.argmax(anomaly1_2) + 72
Smin1_2 = np.argmin(anomaly1_2) + 72

anomaly1_3 = anomaly1[141:216]
Smax1_3 = np.argmax(anomaly1_3) + 141
Smin1_3 = np.argmin(anomaly1_3) + 141

anomaly1_4 = anomaly1[217:280]
Smax1_4 = np.argmax(anomaly1_4) + 217
Smin1_4 = np.argmin(anomaly1_4) + 217

anomaly1_5 = anomaly1[281:352]
Smax1_5 = np.argmax(anomaly1_5) + 281
Smin1_5 = np.argmin(anomaly1_5) + 281

anomaly1_6 = anomaly1[353:424]
Smax1_6 = np.argmax(anomaly1_6) + 353
Smin1_6 = np.argmin(anomaly1_6) + 353

anomaly1_7 = anomaly1[425:496]
Smax1_7 = np.argmax(anomaly1_7) + 425
Smin1_7 = np.argmin(anomaly1_7) + 425

anomaly1_8 = anomaly1[497:544]
Smax1_8 = np.argmax(anomaly1_8) + 497
Smin1_8 = np.argmin(anomaly1_8) + 497

anomaly1_9 = anomaly1[545:592]
Smax1_9 = np.argmax(anomaly1_9) + 545
Smin1_9 = np.argmin(anomaly1_9) + 545

anomaly1_10 = anomaly1[593:640]
Smax1_10 = np.argmax(anomaly1_10) + 593
Smin1_10 = np.argmin(anomaly1_10) + 593

anomaly1_11 = anomaly1[641:688]
```

```
Smax1_11 = np.argmax(anomaly1_11) + 641
Smin1_11 = np.argmin(anomaly1_11) + 641

anomaly1_12 = anomaly1[689:784]
Smax1_12 = np.argmax(anomaly1_12) + 689
Smin1_12 = np.argmin(anomaly1_12) + 689

anomaly1_13 = anomaly1[785:832]
Smax1_13 = np.argmax(anomaly1_13) + 785
Smin1_13 = np.argmin(anomaly1_13) + 785

anomaly1_14 = anomaly1[833:900]
Smax1_14 = np.argmax(anomaly1_14) + 833
Smin1_14 = np.argmin(anomaly1_14) + 833

minmax1 = [
    [anomaly1[Smax1_1], S_[Smax1_1], anomaly1[Smin1_1], S_[Smin1_1]],
    [anomaly1[Smax1_2], S_[Smax1_2], anomaly1[Smin1_2], S_[Smin1_2]],
    [anomaly1[Smax1_3], S_[Smax1_3], anomaly1[Smin1_3], S_[Smin1_3]],
    [anomaly1[Smax1_4], S_[Smax1_4], anomaly1[Smin1_4], S_[Smin1_4]],
    [anomaly1[Smax1_5], S_[Smax1_5], anomaly1[Smin1_5], S_[Smin1_5]],
    [anomaly1[Smax1_6], S_[Smax1_6], anomaly1[Smin1_6], S_[Smin1_6]],
    [anomaly1[Smax1_7], S_[Smax1_7], anomaly1[Smin1_7], S_[Smin1_7]],
    [anomaly1[Smax1_8], S_[Smax1_8], anomaly1[Smin1_8], S_[Smin1_8]],
    [anomaly1[Smax1_9], S_[Smax1_9], anomaly1[Smin1_9], S_[Smin1_9]],
    [anomaly1[Smax1_10], S_[Smax1_10], anomaly1[Smin1_10], S_[Smin1_10]],
    [anomaly1[Smax1_11], S_[Smax1_11], anomaly1[Smin1_11], S_[Smin1_11]],
    [anomaly1[Smax1_12], S_[Smax1_12], anomaly1[Smin1_12], S_[Smin1_12]],
    [anomaly1[Smax1_13], S_[Smax1_13], anomaly1[Smin1_13], S_[Smin1_13]],
    [anomaly1[Smax1_14], S_[Smax1_14], anomaly1[Smin1_14], S_[Smin1_14]]
]
print('MAY 6 TO MAY 22 ACCUMLATED ENERGY MAXIMUMS AND MINIMUMS')
print()
print('{:<12}  {:<16} {:<12} {:<16}'.format('Max J/m2','Max Position m', 'Min J/m2','Min Position
m'))
for v in minmax1:
    maxval, maxpos, minval, minpos = v
    print('{0:<12.3E}  {1:<16.5} {2:<12.3E} {3:<16.5}'.format(maxval, maxpos, minval, minpos))
print()

minmax2 = [
    [anomaly2[Smax1_1], S_[Smax1_1], anomaly2[Smin1_1], S_[Smin1_1]],
    [anomaly2[Smax1_2], S_[Smax1_2], anomaly2[Smin1_2], S_[Smin1_2]],
    [anomaly2[Smax1_3], S_[Smax1_3], anomaly2[Smin1_3], S_[Smin1_3]],
    [anomaly2[Smax1_4], S_[Smax1_4], anomaly2[Smin1_4], S_[Smin1_4]],
    [anomaly2[Smax1_5], S_[Smax1_5], anomaly2[Smin1_5], S_[Smin1_5]],
    [anomaly2[Smax1_6], S_[Smax1_6], anomaly2[Smin1_6], S_[Smin1_6]],
    [anomaly2[Smax1_7], S_[Smax1_7], anomaly2[Smin1_7], S_[Smin1_7]],
    [anomaly2[Smax1_8], S_[Smax1_8], anomaly2[Smin1_8], S_[Smin1_8]],
    [anomaly2[Smax1_9], S_[Smax1_9], anomaly2[Smin1_9], S_[Smin1_9]],
    [anomaly2[Smax1_10], S_[Smax1_10], anomaly2[Smin1_10], S_[Smin1_10]],
    [anomaly2[Smax1_11], S_[Smax1_11], anomaly2[Smin1_11], S_[Smin1_11]],
    [anomaly2[Smax1_12], S_[Smax1_12], anomaly2[Smin1_12], S_[Smin1_12]],
    [anomaly2[Smax1_13], S_[Smax1_13], anomaly2[Smin1_13], S_[Smin1_13]],
    [anomaly2[Smax1_14], S_[Smax1_14], anomaly2[Smin1_14], S_[Smin1_14]]
]
print('JUNE 13 TO JULY 9 ACCUMLATED ENERGY MAXIMUMS AND MINIMUMS')
print()
print('{:<12}  {:<16} {:<12} {:<16}'.format('Max J/m2','Max Position m', 'Min J/m2','Min Position
m'))
for v in minmax2:
    maxval, maxpos, minval, minpos = v
    print('{0:<12.3E}  {1:<16.5} {2:<12.3E} {3:<16.5}'.format(maxval, maxpos, minval, minpos))

print()

minmax3 = [
    [anomaly3[Smax1_1], S_[Smax1_1], anomaly3[Smin1_1], S_[Smin1_1]],
    [anomaly3[Smax1_2], S_[Smax1_2], anomaly3[Smin1_2], S_[Smin1_2]],
```

```
    [anomaly3[Smax1_3], S_[Smax1_3], anomaly3[Smin1_3], S_[Smin1_3]],
    [anomaly3[Smax1_4], S_[Smax1_4], anomaly3[Smin1_4], S_[Smin1_4]],
    [anomaly3[Smax1_5], S_[Smax1_5], anomaly3[Smin1_5], S_[Smin1_5]],
    [anomaly3[Smax1_6], S_[Smax1_6], anomaly3[Smin1_6], S_[Smin1_6]],
    [anomaly3[Smax1_7], S_[Smax1_7], anomaly3[Smin1_7], S_[Smin1_7]],
    [anomaly3[Smax1_8], S_[Smax1_8], anomaly3[Smin1_8], S_[Smin1_8]],
    [anomaly3[Smax1_9], S_[Smax1_9], anomaly3[Smin1_9], S_[Smin1_9]],
    [anomaly3[Smax1_10], S_[Smax1_10], anomaly3[Smin1_10], S_[Smin1_10]],
    [anomaly3[Smax1_11], S_[Smax1_11], anomaly3[Smin1_11], S_[Smin1_11]],
    [anomaly3[Smax1_12], S_[Smax1_12], anomaly3[Smin1_12], S_[Smin1_12]],
    [anomaly3[Smax1_13], S_[Smax1_13], anomaly3[Smin1_13], S_[Smin1_13]],
    [anomaly3[Smax1_14], S_[Smax1_14], anomaly3[Smin1_14], S_[Smin1_14]]
]
print('JULY 13 TO AUGUST 28 ACCUMLATED ENERGY MAXIMUMS AND MINIMUMS')
print()
print('{:<12}  {:<16} {:<12} {:<16}'.format('Max J/m2','Max Position m', 'Min J/m2','Min Position
m'))
for v in minmax3:
    maxval, maxpos, minval, minpos = v
    print('{0:<12.3E}  {1:<16.5} {2:<12.3E} {3:<16.5}'.format(maxval, maxpos, minval, minpos))
print()
```

```
MAY 6 TO MAY 22 ACCUMLATED ENERGY MAXIMUMS AND MINIMUMS

Max J/m2      Max Position m   Min J/m2     Min Position m
2.440E+08     4.0612           3.418E+07    0.53437
3.086E+08     10.26            1.567E+08    7.6949
3.371E+08     22.657           1.976E+08    19.344
3.414E+08     28.856           2.034E+08    25.543
3.440E+08     35.054           2.069E+08    31.741
3.460E+08     41.253           2.093E+08    37.94
3.475E+08     47.452           2.126E+08    50.337
3.488E+08     53.65            2.139E+08    56.536
3.500E+08     59.849           2.154E+08    62.734
3.514E+08     66.048           2.259E+08    63.376
3.533E+08     72.246           2.171E+08    68.933
4.790E+08     82.613           2.195E+08    75.132
4.790E+08     88.812           2.604E+08    84.537
4.776E+08     89.025           1.187E+08    95.758

JUNE 13 TO JULY 9 ACCUMLATED ENERGY MAXIMUMS AND MINIMUMS

Max J/m2      Max Position m   Min J/m2     Min Position m
4.381E+08     4.0612           6.738E+07    0.53437
5.533E+08     10.26            2.873E+08    7.6949
6.041E+08     22.657           3.597E+08    19.344
6.117E+08     28.856           3.701E+08    25.543
6.165E+08     35.054           3.764E+08    31.741
6.199E+08     41.253           3.806E+08    37.94
6.226E+08     47.452           3.864E+08    50.337
6.248E+08     53.65            3.888E+08    56.536
6.271E+08     59.849           3.913E+08    62.734
6.295E+08     66.048           4.103E+08    63.376
6.329E+08     72.246           3.942E+08    68.933
8.510E+08     82.613           3.985E+08    75.132
8.511E+08     88.812           4.674E+08    84.537
8.485E+08     89.025           2.120E+08    95.758

JULY 13 TO AUGUST 28 ACCUMLATED ENERGY MAXIMUMS AND MINIMUMS

Max J/m2      Max Position m   Min J/m2     Min Position m
7.434E+08     4.0612           1.062E+08    0.53437
9.399E+08     10.26            4.795E+08    7.6949
1.027E+09     22.657           6.037E+08    19.344
1.040E+09     28.856           6.215E+08    25.543
1.048E+09     35.054           6.322E+08    31.741
1.054E+09     41.253           6.394E+08    37.94
1.058E+09     47.452           6.493E+08    50.337
1.062E+09     53.65            6.534E+08    56.536
1.066E+09     59.849           6.577E+08    62.734
1.070E+09     66.048           6.898E+08    63.376
1.076E+09     72.246           6.629E+08    68.933
1.456E+09     82.613           6.704E+08    75.132
1.456E+09     88.812           7.932E+08    84.537
1.452E+09     89.025           3.611E+08    95.758

CPU times: user 834 µs, sys: 309 µs, total: 1.14 ms
Wall time: 928 µs
```

In [65]:

```
%%time

evap_anomaly1_1 = evap_anomaly1[0:71]
evap_Smax1_1 = np.argmax(evap_anomaly1_1)
evap_Smin1_1 = np.argmin(evap_anomaly1_1)

evap_anomaly1_2 = evap_anomaly1[72:140]
evap_Smax1_2 = np.argmax(evap_anomaly1_2) + 72
evap_Smin1_2 = np.argmin(evap_anomaly1_2) + 72

evap_anomaly1_3 = evap_anomaly1[141:216]
```

```
evap_Smax1_3 = np.argmax(evap_anomaly1_3) + 141
evap_Smin1_3 = np.argmin(evap_anomaly1_3) + 141

evap_anomaly1_4 = evap_anomaly1[217:280]
evap_Smax1_4 = np.argmax(evap_anomaly1_4) + 217
evap_Smin1_4 = np.argmin(evap_anomaly1_4) + 217

evap_anomaly1_5 = evap_anomaly1[281:352]
evap_Smax1_5 = np.argmax(evap_anomaly1_5) + 281
evap_Smin1_5 = np.argmin(evap_anomaly1_5) + 281

evap_anomaly1_6 = evap_anomaly1[353:424]
evap_Smax1_6 = np.argmax(evap_anomaly1_6) + 353
evap_Smin1_6 = np.argmin(evap_anomaly1_6) + 353

evap_anomaly1_7 = evap_anomaly1[425:496]
evap_Smax1_7 = np.argmax(evap_anomaly1_7) + 425
evap_Smin1_7 = np.argmin(evap_anomaly1_7) + 425

evap_anomaly1_8 = evap_anomaly1[497:544]
evap_Smax1_8 = np.argmax(evap_anomaly1_8) + 497
evap_Smin1_8 = np.argmin(evap_anomaly1_8) + 497

evap_anomaly1_9 = evap_anomaly1[545:592]
evap_Smax1_9 = np.argmax(evap_anomaly1_9) + 545
evap_Smin1_9 = np.argmin(evap_anomaly1_9) + 545

evap_anomaly1_10 = evap_anomaly1[593:640]
evap_Smax1_10 = np.argmax(evap_anomaly1_10) + 593
evap_Smin1_10 = np.argmin(evap_anomaly1_10) + 593

evap_anomaly1_11 = evap_anomaly1[641:688]
evap_Smax1_11 = np.argmax(evap_anomaly1_11) + 641
evap_Smin1_11 = np.argmin(evap_anomaly1_11) + 641

evap_anomaly1_12 = evap_anomaly1[689:784]
evap_Smax1_12 = np.argmax(evap_anomaly1_12) + 689
evap_Smin1_12 = np.argmin(evap_anomaly1_12) + 689

evap_anomaly1_13 = evap_anomaly1[785:832]
evap_Smax1_13 = np.argmax(evap_anomaly1_13) + 785
evap_Smin1_13 = np.argmin(evap_anomaly1_13) + 785

evap_anomaly1_14 = evap_anomaly1[833:900]
evap_Smax1_14 = np.argmax(evap_anomaly1_14) + 833
evap_Smin1_14 = np.argmin(evap_anomaly1_14) + 833

evap_minmax1 = [
    [evap_anomaly1[evap_Smax1_1], S_[evap_Smax1_1], evap_anomaly1[evap_Smin1_1], S_[evap_Smin1_1]
],
    [evap_anomaly1[evap_Smax1_2], S_[evap_Smax1_2], evap_anomaly1[evap_Smin1_2], S_[evap_Smin1_2]
],
    [evap_anomaly1[evap_Smax1_3], S_[evap_Smax1_3], evap_anomaly1[evap_Smin1_3], S_[evap_Smin1_3]
],
    [evap_anomaly1[evap_Smax1_4], S_[evap_Smax1_4], evap_anomaly1[evap_Smin1_4], S_[evap_Smin1_4]
],
    [evap_anomaly1[evap_Smax1_5], S_[evap_Smax1_5], evap_anomaly1[evap_Smin1_5], S_[evap_Smin1_5]
],
    [evap_anomaly1[evap_Smax1_6], S_[evap_Smax1_6], evap_anomaly1[evap_Smin1_6], S_[evap_Smin1_6]
],
    [evap_anomaly1[evap_Smax1_7], S_[evap_Smax1_7], evap_anomaly1[evap_Smin1_7], S_[evap_Smin1_7]
],
    [evap_anomaly1[evap_Smax1_8], S_[evap_Smax1_8], evap_anomaly1[evap_Smin1_8], S_[evap_Smin1_8]
],
    [evap_anomaly1[evap_Smax1_9], S_[evap_Smax1_9], evap_anomaly1[evap_Smin1_9], S_[evap_Smin1_9]
],
    [evap_anomaly1[evap_Smax1_10], S_[evap_Smax1_10], evap_anomaly1[evap_Smin1_10], S_[evap_Smin1
_10]],
    [evap_anomaly1[evap_Smax1_11], S_[evap_Smax1_11], evap_anomaly1[evap_Smin1_11], S_[evap_Smin1
```

```
_11]],
    [evap_anomaly1[evap_Smax1_12], S_[evap_Smax1_12], evap_anomaly1[evap_Smin1_12], S_[evap_Smin1
_12]],
    [evap_anomaly1[evap_Smax1_13], S_[evap_Smax1_13], evap_anomaly1[evap_Smin1_13], S_[evap_Smin1
_13]],
    [evap_anomaly1[evap_Smax1_14], S_[evap_Smax1_14], evap_anomaly1[evap_Smin1_14], S_[evap_Smin1
_14]]
]
print('MAY 6 TO MAY 22 ACCUMLATED EVAPORATION POTENTIAL MAXIMUMS AND MINIMUMS')
print()
print('{:<12}  {:<16} {:<12} {:<16}'.format('Max cm','Max Position m', 'Min cm','Min Position m')
)
for evap_v in evap_minmax1:
    evap_maxval, evap_maxpos, evap_minval, evap_minpos = evap_v
    print('{0:<12.3f}  {1:<16.5} {2:<12.3f} {3:<16.5}'.format(evap_maxval, evap_maxpos, evap_minv
al, evap_minpos))
print()

evap_minmax2 = [
    [evap_anomaly2[evap_Smax1_1], S_[evap_Smax1_1], evap_anomaly2[evap_Smin1_1], S_[evap_Smin1_1]
],
    [evap_anomaly2[evap_Smax1_2], S_[evap_Smax1_2], evap_anomaly2[evap_Smin1_2], S_[evap_Smin1_2]
],
    [evap_anomaly2[evap_Smax1_3], S_[evap_Smax1_3], evap_anomaly2[evap_Smin1_3], S_[evap_Smin1_3]
],
    [evap_anomaly2[evap_Smax1_4], S_[evap_Smax1_4], evap_anomaly2[evap_Smin1_4], S_[evap_Smin1_4]
],
    [evap_anomaly2[evap_Smax1_5], S_[evap_Smax1_5], evap_anomaly2[evap_Smin1_5], S_[evap_Smin1_5]
],
    [evap_anomaly2[evap_Smax1_6], S_[evap_Smax1_6], evap_anomaly2[evap_Smin1_6], S_[evap_Smin1_6]
],
    [evap_anomaly2[evap_Smax1_7], S_[evap_Smax1_7], evap_anomaly2[evap_Smin1_7], S_[evap_Smin1_7]
],
    [evap_anomaly2[evap_Smax1_8], S_[evap_Smax1_8], evap_anomaly2[evap_Smin1_8], S_[evap_Smin1_8]
],
    [evap_anomaly2[evap_Smax1_9], S_[evap_Smax1_9], evap_anomaly2[evap_Smin1_9], S_[evap_Smin1_9]
],
    [evap_anomaly2[evap_Smax1_10], S_[evap_Smax1_10], evap_anomaly2[evap_Smin1_10], S_[evap_Smin1
_10]],
    [evap_anomaly2[evap_Smax1_11], S_[evap_Smax1_11], evap_anomaly2[evap_Smin1_11], S_[evap_Smin1
_11]],
    [evap_anomaly2[evap_Smax1_12], S_[evap_Smax1_12], evap_anomaly2[evap_Smin1_12], S_[evap_Smin1
_12]],
    [evap_anomaly2[evap_Smax1_13], S_[evap_Smax1_13], evap_anomaly2[evap_Smin1_13], S_[evap_Smin1
_13]],
    [evap_anomaly2[evap_Smax1_14], S_[evap_Smax1_14], evap_anomaly2[evap_Smin1_14], S_[evap_Smin1
_14]]
]
print('JUNE 13 TO JULY 9 ACCUMLATED EVAPORATION POTENTIAL MAXIMUMS AND MINIMUMS')
print()
print('{:<12}  {:<16} {:<12} {:<16}'.format('Max cm','Max Position m', 'Min cm','Min Position m')
)
for evap_v in evap_minmax2:
    evap_maxval, evap_maxpos, evap_minval, evap_minpos = evap_v
    print('{0:<12.3f}  {1:<16.5} {2:<12.3f} {3:<16.5}'.format(evap_maxval, evap_maxpos, evap_minv
al, evap_minpos))

print()

evap_minmax3 = [
    [evap_anomaly3[evap_Smax1_1], S_[evap_Smax1_1], evap_anomaly3[evap_Smin1_1], S_[evap_Smin1_1]
],
    [evap_anomaly3[evap_Smax1_2], S_[evap_Smax1_2], evap_anomaly3[evap_Smin1_2], S_[evap_Smin1_2]
],
    [evap_anomaly3[evap_Smax1_3], S_[evap_Smax1_3], evap_anomaly3[evap_Smin1_3], S_[evap_Smin1_3]
],
    [evap_anomaly3[evap_Smax1_4], S_[evap_Smax1_4], evap_anomaly3[evap_Smin1_4], S_[evap_Smin1_4]
],
    [evap_anomaly3[evap_Smax1_5], S_[evap_Smax1_5], evap_anomaly3[evap_Smin1_5], S_[evap_Smin1_5]
```

```
],
    [evap_anomaly3[evap_Smax1_6], S_[evap_Smax1_6], evap_anomaly3[evap_Smin1_6], S_[evap_Smin1_6]
],
    [evap_anomaly3[evap_Smax1_7], S_[evap_Smax1_7], evap_anomaly3[evap_Smin1_7], S_[evap_Smin1_7]
],
    [evap_anomaly3[evap_Smax1_8], S_[evap_Smax1_8], evap_anomaly3[evap_Smin1_8], S_[evap_Smin1_8]
],
    [evap_anomaly3[evap_Smax1_9], S_[evap_Smax1_9], evap_anomaly3[evap_Smin1_9], S_[evap_Smin1_9]
],
    [evap_anomaly3[evap_Smax1_10], S_[evap_Smax1_10], evap_anomaly3[evap_Smin1_10], S_[evap_Smin1
_10]],
    [evap_anomaly3[evap_Smax1_11], S_[evap_Smax1_11], evap_anomaly3[evap_Smin1_11], S_[evap_Smin1
_11]],
    [evap_anomaly3[evap_Smax1_12], S_[evap_Smax1_12], evap_anomaly3[evap_Smin1_12], S_[evap_Smin1
_12]],
    [evap_anomaly3[evap_Smax1_13], S_[evap_Smax1_13], evap_anomaly3[evap_Smin1_13], S_[evap_Smin1
_13]],
    [evap_anomaly3[evap_Smax1_14], S_[evap_Smax1_14], evap_anomaly3[evap_Smin1_14], S_[evap_Smin1
_14]]
]
print('JULY 13 TO AUGUST 28 ACCUMLATED EVAPORATION POTENTIAL MAXIMUMS AND MINIMUMS')
print()
print('{:<12}  {:<16} {:<12} {:<16}'.format('Max cm','Max Position m', 'Min cm','Min Position m')
)
for evap_v in evap_minmax3:
    evap_maxval, evap_maxpos, evap_minval, evap_minpos = evap_v
    print('{0:<12.3f}  {1:<16.5} {2:<12.3f} {3:<16.5}'.format(evap_maxval, evap_maxpos, evap_minv
al, evap_minpos))
print()
```

MAY 6 TO MAY 22 ACCUMLATED EVAPORATION POTENTIAL MAXIMUMS AND MINIMUMS

Max cm	Max Position m	Min cm	Min Position m
9.982	4.0612	1.398	0.53437
12.623	10.26	6.411	7.6949
13.791	22.657	8.083	19.344
13.965	28.856	8.323	25.543
14.074	35.054	8.466	31.741
14.153	41.253	8.564	37.94
14.215	47.452	8.697	50.337
14.267	53.65	8.752	56.536
14.319	59.849	8.810	62.734
14.376	66.048	9.240	63.376
14.454	72.246	8.880	68.933
19.596	82.613	8.981	75.132
19.597	88.812	10.652	84.537
19.539	89.025	4.854	95.758

JUNE 13 TO JULY 9 ACCUMLATED EVAPORATION POTENTIAL MAXIMUMS AND MINIMUMS

Max cm	Max Position m	Min cm	Min Position m
17.923	4.0612	2.757	0.53437
22.634	10.26	11.752	7.6949
24.715	22.657	14.714	19.344
25.024	28.856	15.141	25.543
25.219	35.054	15.397	31.741
25.359	41.253	15.570	37.94
25.468	47.452	15.807	50.337
25.562	53.65	15.904	56.536
25.653	59.849	16.006	62.734
25.754	66.048	16.786	63.376
25.890	72.246	16.128	68.933
34.815	82.613	16.304	75.132
34.816	88.812	19.121	84.537
34.711	89.025	8.673	95.758

JULY 13 TO AUGUST 28 ACCUMLATED EVAPORATION POTENTIAL MAXIMUMS AND MINIMUMS

Max cm	Max Position m	Min cm	Min Position m
30.412	4.0612	4.346	0.53437
38.450	10.26	19.616	7.6949
42.002	22.657	24.695	19.344
42.530	28.856	25.425	25.543
42.863	35.054	25.862	31.741
43.102	41.253	26.158	37.94
43.289	47.452	26.563	50.337
43.449	53.65	26.731	56.536
43.606	59.849	26.908	62.734
43.780	66.048	28.220	63.376
44.016	72.246	27.119	68.933
59.576	82.613	27.424	75.132
59.578	88.812	32.451	84.537
59.401	89.025	14.774	95.758

CPU times: user 754 μs, sys: 435 μs, total: 1.19 ms
Wall time: 809 μs

In [66]:

```
%%time
# Longwavetotal = 2D array across space and time containing lw from panels and sky

SaveArray(longwavetotal, c='overwrite', filename='lw_total1.txt')  # Save values from first time chunk
SaveArray(longwavetotal2, c='overwrite', filename='lw_total2.txt') # Save values from second time chunk
SaveArray(longwavetotal3, c='overwrite', filename='lw_total3.txt') # Save values from third time chunk

filename1 = 'lw_total_1.txt'
LineBreak(c='overwrite', filename=filename1)
for i in range(0,225):
    for j in range(0,1580):
        SaveArray(data=longwavetotal[j,i], c='append', filename=filename1)
    LineBreak(c='append', filename=filename1)
```

```
CPU times: user 28.5 s, sys: 16.5 s, total: 45 s
Wall time: 45.3 s
```

In [67]:

```
%%time

filename2 = 'lw_total_2.txt'
LineBreak(c='overwrite', filename=filename2)
for i in range(0,225):
    for j in range(0,2452):
        SaveArray(longwavetotal2[j,i], c='append', filename=filename2)
    LineBreak(c='append', filename=filename2)
```

```
CPU times: user 43.7 s, sys: 23 s, total: 1min 6s
Wall time: 1min 7s
```

In [68]:

```
%%time

filename3 = 'lw_total_3.txt'
LineBreak(c='overwrite', filename=filename3)
for i in range(0,225):
    for j in range(0,4348):
        SaveArray(longwavetotal3[j,i], c='append', filename=filename3)
    LineBreak(c='append', filename=filename3)
```

```
CPU times: user 1min 18s, sys: 43.4 s, total: 2min 1s
Wall time: 2min 3s
```

In [69]:

```
%%time

for t in range(0,len(times)):
    SaveArray(Lsky[t], c='append', filename='lw_sky.txt')
```

```
CPU times: user 845 ms, sys: 451 ms, total: 1.3 s
Wall time: 1.31 s
```

In [70]:

```
%%time

for t in range(0,len(times)):
    SaveArray(T_panel_adjusted[t], c='append', filename='paneltemps.txt')
```

```
CPU times: user 858 ms, sys: 451 ms, total: 1.31 s
Wall time: 1.33 s
```

In [ ]:
