# Supplementary material for: Agrivoltaics: Modeling the relative importance of longwave radiation from solar panels
Source: PLoS One. 2022 Oct 28;17(10):e0273119. doi: 10.1371/journal.pone.0273119 (PMC9616218; doi:10.1371/journal.pone.0273119)
Supplement: Attachment — Submitted filename: Response to Reviewers.docx [file pone.0273119.s002.docx]

Thank you for your thoughtful comments. We have responded to each item below in italics.

Reviewer #1: The work is logically structured and consequential in its exposition. The article develops a model to quantify the downwelling longwave energy at the ground to explore the soil moisture loss under the time of 2018. I cannot recommend considering this paper for publication before major modifications and change. My comments are as follows:
1. The order result of uploaded image is a mess. In addition, the horizontal and vertical coordinates are not clear in Fig. 3.

*Thank you, the figures have been remade.*

2. The missing weather parameters for small time steps (less than 1 day) were approximated via interpolation in this paper, but the larger time steps (more than 1 day)?

*For gaps in the weather data greater than 1 day, interpolation would not have reflected the true weather patterns. Therefore, the longwave radiative heat flux was not estimated for these gaps, which resulted in three separate time spans for which the model was executed. I already specify this in the section titled ‘Flux Over Time: Assimilating with Spatial Data.’*

3. The clear skies condition is assumed in modeling work, whether the proposed model can be set up under more complex environmental conditions (cloudy sky, cloudy in the morning and clear sky in the afternoon). All measured data was collected in the clear skies in the model? Please provide detail explain the applicability and advantages of model.

*Thank you for your comment. We have added the following to the conclusions:*

*However, the clear skies assumption is the case where longwave radiation has the minimum contribution to the energy balance. That is, high shortwave radiation and low downwelling longwave radiation. Note that in Fig. 14, the longwave varies from ~5% to 70% of the total incoming radiation. Therefore, even in this case, it cannot be ignored.*

4. Two long straight lines describe the interpolated values as shown in Fig. 10a, the first straight line shows a long time. However, it shows a volatility change, how to verify the rationality of a long time interpolation, data consistency or data series test?

*This was merely an artifact of the plotting, but was not used in the analysis. Fig. 10a has been remade to reflect this.*

5. The frequencies of panel temperatures show an obvious frequency difference in Fig. 10b, please explain this change.

*Very high temperatures are less common than the mean or median temperatures.*

6. In the work I do not see any mention about model both parameter calibration and validation. A model is applied in this study, it is suggested that these differences of simulated and measured result can be emphasized the quantified analysis.

*Our contribution in this manuscript is the longwave radiation model. Parameters, data, physical constants, independent and dependent variables for this model are defined in Tables 1 and 2. The values presented in Table 1 are direct measurements from our case study. All of the angles in Table 2 are intermediate values held in the trigonometry (pure math). The emissivity was assumed to be 1 (black body). However, if you consider Eqn. 4, the flux is proportional to the emissivity. Therefore, a 1% change in emissivity would result in a 1% change in the flux. All remaining equations (including the shortwave radiation model) were established models in the literature.*

7. I believe that the Authors should do more work. The study analyzes the difference and change of in longwave energy, and another approximation of downwelling longwave radiation in the model is computed. It should provide the more discusses and analysis for the effect of longwave radiation on soil moisture.

*We agree that additional analysis was warranted. The most significant change we made was shifting the focus of the paper to predicting spatial variability in energy rather than soil moisture. There are too many other contributors to soil moisture to meaningfully predict it with only this model. However, rather than expanding upon soil moisture, we chose to incorporate incoming shortwave radiation into the model, as requested by Reviewer #2. The shortwave radiative heat flux under the solar panels was computed and added to the longwave radiation to yield the total radiative heat flux at the land surface. This brings us one step closer to the total energy budget available to the system.*

Reviewer #2: This is a interesting research dealing with the calculation of the long-wave radiation flux induced by the solar panels. The authors presented detailed and analytical geometrical schemes and calculating procedures, which provide very good insights for future research.

*Thank you for your commentary and your effort in reviewing this manuscript.*

However, the problem is that, are the data of calculated long-wave radiation flux right? why the evaporation contributed by it could be 60-80 cm, and you admitted that the value was too high. There might be something wrong in calculation the radiation, or the calculation of evaporation.

*Given your comments and the comments of the other reviewer, we believe that the soil moisture units distracted from the main point of the manuscript. Thus, we refocused the results, discussions, and conclusions on the radiation itself; in particular, the variability of that radiation. Refer to the new figures 13 and 14 for these details. Our decision to focus on shortwave radiation was inspired by your comment, as it provided needed context for the relative magnitudes of fluxes, and increases confidence in model results. Note that the additional longwave radiation in the open aisles backfills the energetic reduction of the shortwave. We also added a section that discusses the potential extreme values for our models, and all our results fall within these physical limitations.*

I expect more solid and useful results, that should be, your calculation of the ground intercepted radiation and ground soil evaporation are all validated by the field measurements. Both long and short waves should be considered.

*We agree that a comparison with field data is a valuable next step. However, we chose to focus this mathematically dense manuscript on model development. Shortwave has been added per your suggestion.*

Sincerely,

Laurel Shepard

Chad Higgins
